# A novel drip-irrigative technique for enhanced epiretinal perfluorocarbon liquid clearance during vitreoretinal surgery

**DOI:** 10.1186/s40942-024-00591-z

**Published:** 2024-10-11

**Authors:** Jingli Guo, Victoria Y. Gu, Yuhan Zhou, Peiquan Zhao, Dongsheng Zhao

**Affiliations:** 1 Department of Ophthalmology, Xin Hua Hospital, Shanghai Jiao Tong University School of Medicine, Shanghai, China; 2 Johns Hopkins Bloomberg School of Public Health, Baltimore, MD USA; 3 Fudan University, Shanghai, China

**Keywords:** Perfluorocarbon liquid, Basic salt solution, PFCL clearance, Rhegmatogenous retinal detachment, Subretinal PFCL

## Abstract

**Purpose:**

To present a novel intraoperative application technique of basic salt solution (BSS) perfusate to address residual epiretinal perfluorocarbon liquid (PFCL) droplets.

**Methods:**

Following standard liquid-gas exchange and aspiration of visible PFCL using a flute needle, the adjuvant drip-irrigative method is employed. A 2mL needle containing BSS is introduced and maneuvered circumferentially around the posterior pole while injecting BSS intermittently to obviate droplet presence. Subsequently, droplets lying flat to the surface drain via the flute needle, and the process if repeated until no droplets are visible.

**Results:**

Among 112 consecutive patients diagnosed with rhegmatogenous retinal detachment (RDD) with at least 3 months follow-up, 109 patients (109 eyes, [97%]) experienced no PFCL-related complications follow pars plana vitrectomy. Among three patients with PFCL-related complications, two (2 eyes) presented with residual droplets on the retinal surface during silicone oil retrieval, and one (1 eyes) had PFCL migration to the anterior chamber. No patients experienced sub-retinal/ sub-foveal PFCL or iatrogenic injury.

**Conclusion:**

This adjuvant drip-irrigative technique offers enhanced droplet visibility, reduced risk of iatrogenic retinal damage, and ease of application. Findings reported suggest the potential of this approach as a standard practice when using PFCL to mitigate complications.

**Supplementary Information:**

The online version contains supplementary material available at 10.1186/s40942-024-00591-z.

## Key summary point

This study introduces a drip-irrigative technique using a novel saline application method to enhance visibility and drainage of residual droplets during surgery, thereby reducing the risk of iatrogenic damage and migration. A case series supports its effectiveness, highlighting its potential as a safe adjuvant practice for vitreoretinal surgeons.

**Supplementary Information:**

The online version contains supplementary material available at 10.1186/s40942-024-00591-z.

Perfluorocarbon liquid (PFCL) is frequently used as a temporary tamponade during vitreoretinal surgery for retinal tears, retinopathies, and ocular trauma. Its physical properties include a specific gravity above water, transparency for visualization, low viscosity for ease of injection, high boiling point to facilitate direct or photocoagulation and prevent vaporization, low surface tension, and high interfacial tension [1]. PFCL is also immiscible with blood, water, and silicone oil, thus aiding in situations of direct compression and hemostasis and allowing for its mobilization of the crystal nucleus and foreign matter for easier removal [2]. Thoughtful management of these physical and chemical properties is critical for surgeons to fully utilize its advantages and avoid complications.

Despite these advantages, PFCL’s biocompatibility yields challenges: retention and migration can cause photoreceptor cell harm, reduced vascular flow, and lesions in the subretinal space, and high intraocular pressure and corneal toxicity in the anterior chamber. Additionally, aggressive aspiration of PFCL during retrieval can cause iatrogenic damage to the fovea and macula [3]. As such, surgeons have the difficult task of aspirating as much visible PFCL as possible, without knowing if some amounts are retained because of the difficulty in visually monitoring caused by refraction. While the literature reports on various methods for postoperative resolution of subretinal PFCL and PFCL droplets in the anterior chamber, with varying degrees of success, few focus on optimizing intraoperative PFCL drainage and even fewer can circumvent iatrogenic retinal damage [3–6]. This article presents a technique of drip-irrigating, or “drip-rinsing”, droplets using basic salt solution (BSS) perfusate to enhance surgical visibility of otherwise refractorily imperceptible deposits. BSS is a fluid medium also commonplace to vitreoretinal surgeons for maintaining intraocular pressure. BSS is less dense than PFCL and more dense than silicone oil; the three tiers of specific gravities yields stratification which facilitates both injection and expression.

## Methods

All patients were operated on by one surgeon (D.S.Z.) who required PFCL-enabled (DK-Line, Bausch + Lomb, Germany) pars plana vitrectomy (PPV) for rhegmatogenous retinal detachment (RRD) using a conventional vitrectomy suite (Alcon, Constellation, Fort Worth, TX). Patients fitting the inclusion criteria were identified from Xinhua Hospital records between February 2023 to December 2023. Metrics of interest included: number of cases in which additional PFCL droplets were observed and aspirated following technique application, length of follow-up, and remnant PFCL-related complications, as observed in outpatient clinic using standard examination, ultrawide-field scanning laser ophthalmoscopy (UWF-SLO) (Optos^®^ PLC, Dunfermline, Scotland, United Kingdom), and optical coherence tomography (OCT) (RTVue-100, Optovue, Inc., Fremont, CA, United States). Remnant PFCL-related complications included sub-retinal/ sub-foveal retention, iatrogenic injury, anterior chamber migration, retinal or choroidal ischemia, intraocular inflammation, emulsification, and visual interference. This study adhered to the tenets of the Declaration of Helsinki and Ethics Committee approval was obtained from the Xinhua Hospital institutional review board. Informed consent was obtained from all patients or their guardians.

## Surgical technique

**Fig. 1:**
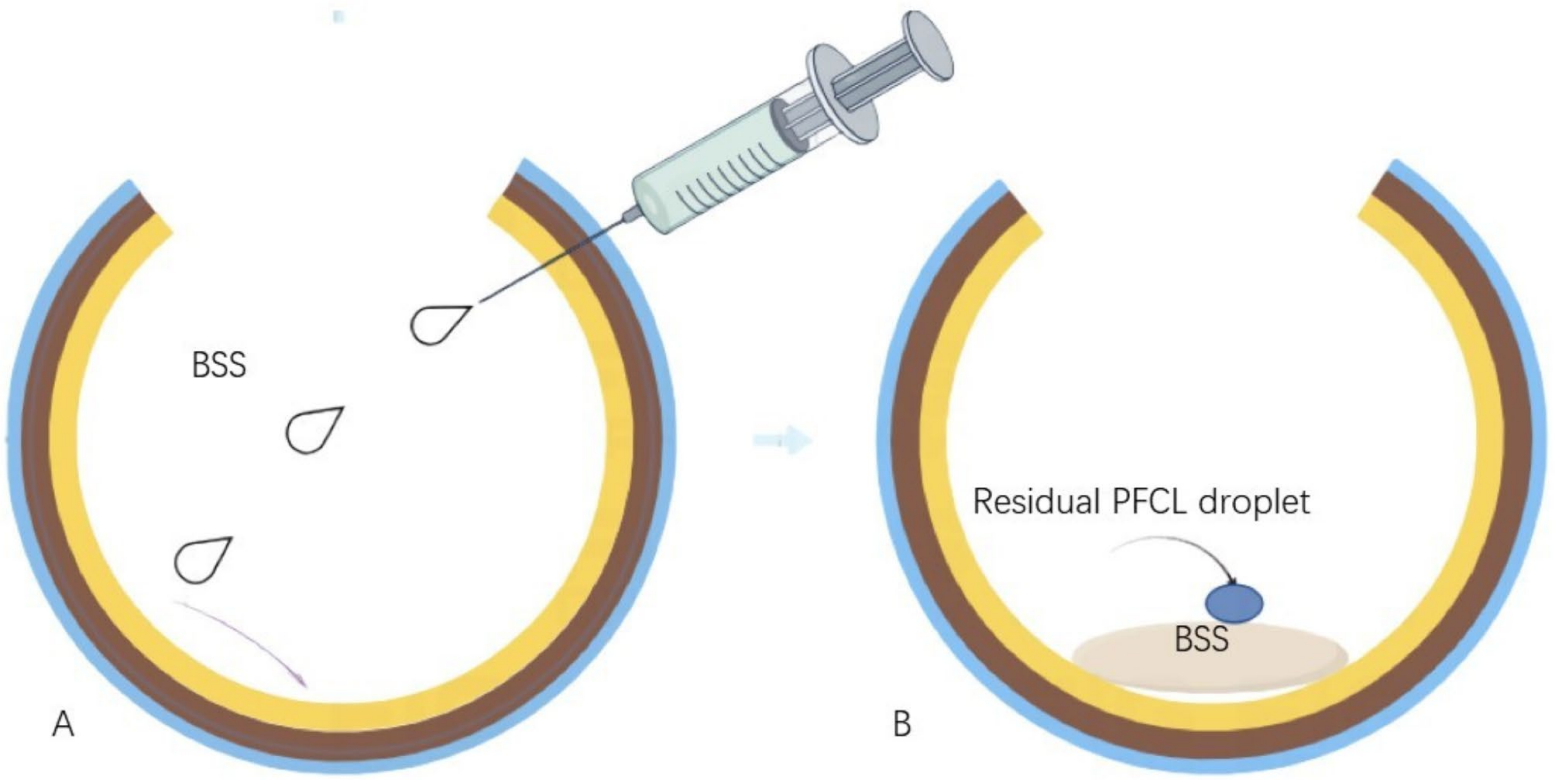
Schematic drawing to show the main steps of the surgical technique used in the present study. **A**: A 2mL needle containing BSS is introduced through the 25-gauge trocar into the vitreous cavity. **B**: Perfluorocarbon liquid droplets begin floating to the surface due to their lower specific gravities and immiscibility and drain via the flute needle

This technique is relevant for clearing epiretinal PFCL during PPV to reduce the likelihood of residual PFCL complications. The process begins with a fluid-air exchange, using a flute needle to aspirate subretinal and epiretinal fluids around the posterior pole, if present, until all PFCL visible is reasonably cleared. To begin the adjuvant drip-irrigation, a 2mL needle containing BSS is introduced through the 23-gauge trocar into the vitreous cavity. After adjusting pressure, the needle is rotated circumferentially around posterior pole to obviate the presence of droplets while intermittently injecting BSS. The sequence of BSS irrigational injections begins from the mid-periphery to first slightly rinse the epiretinal surface and encourage the separation of residual PFCL droplets as well as by the aid of rotating the eye in order to expand the area of the retina. At this point, droplets begin floating to the surface due to their lower specific gravities and immiscibility and drain via the flute needle. This process is repeated in a clockwise motion with 3–8 deposit spots until no droplets are visible (see Supplemental Digital Content 1). The main steps of the surgical technique were shown in Fig. 1.

## Results

### Subjects

One hundred and twelve consecutive patients who were diagnosed with primary (78 eyes) and recurrent uncomplicated (34 eyes) RRD, operated on between February 2023 to December 2023, and had at least 3 months of follow-up were included. Demographic and clinical characteristics were shown in Table 1.

**Table 1:** Demographic and clinical characteristics

Number of patients (eyes)	112 (112)
Sex (Male/Female)	58/54
Age, mean ± SD (range) [years]	46.99 ± 8.94 (12–79)
Baseline Snellen BCVA (range)	20/63 (20/20–20/2000)
Follow-up duration, mean ± SD (range) [months]	6.15 ± 2.82 (4–12)

### Intraoperative procedure

In 109 eyes [97.3%], additional PFCL droplets became visible and were aspirated following the adjuvant BSS rinsing technique. Surgical procedures were recorded by video (Supplementary Video 1). Thirty-seven eyes received drainage retinotomies, and no cases experienced additional intraoperative complications involving PFCL use. Surgical indications and complications were shown Table 2.

**Table 2:** Surgical indications and complications

	Eyes, *n* (%)
PPV indication	
Primary uncomplicated RRD	78 (67)
Recurrent RRD	34 (32)
Received drainage retinotomy	37 (33)
Post-op retained PFCL-related complications (*n* = 3), by location	
Vitreous	2 (1.8)
Anterior Chamber	1 (0.9)
Subretinal	-

PFCL: perfluorocarbon liquid; PPV: pars plana vitrectomy; RRD: rhegmatogenous retinal detachment

### Postoperative observations

After PPV and 6.15 ± 2.82 months of follow-up, one hundred and nine patients (109 eyes) experienced no PFCL-related complications. Three patients (3 eyes) presented with residual PFCL droplets on the retinal surface during silicone oil retrieval, and one patients (1 eyes) had PFCL migration to the anterior chamber during follow-up. These complications resolved with subsequent treatment. No patients experienced sub-retinal/ sub-foveal PFCL, iatrogenic injury, or ischemia.

## Discussion

The true incidence of PFCL-related complications remains unknown, though reported rates have ranged from 1 to 11% for subretinal migration [3]. Current resolution methods for subretinal complications, including cannular puncture and subfoveal BSS injection, can cause scotoma, macular holes, hemorrhage, and choroidal neovascularization and require re-treatment [7]. Others have also previously suggest a limited risk of inflammation from residual droplets, [8] yet evolving understandings of PFCL toxicity may encourage increased caution. As such, the atraumatic mitigation of retained PFCL is of top priority of surgeons. Although some vitreoretinal surgeons have been known to employ BSS perfusate to intraoperatively rinse residual PFCL droplets [9], the adoption rate remains unknown, and the technique is underrepresented in existing literature. Other instances of BSS usage for the displacement and removal of subfoveal PFCL droplets indicate its potential for managing complication yet neglect potential epiretinal and routine applications [6]. To the best of the authors’ knowledge, this study is the first to report on and systematically evaluate the circumferential application of BSS using a flute needle.

This technique’s notable advantages for surgical visibility and safety include:

1. Employment of BSS perfusate to create a distinct liquid level, enhancing PFCL droplet visibility owing to the contrasting specific gravities. Typically, the air bubble causes refraction which obscures residual PFCL.
2. Its circumvention of gas filling in the vitreous cavity, which can lead to refraction or scattering and consequently obscuring heavy water residues.
3. Its use of a more discrete visual indicator, perhaps advantageous for younger surgeons or those with less threshold experience for clearance.
4. Its use as a prophylactic measure for droplet draining, with a low time cost and shallow learning curve.
5. Its mitigation of scenarios involving high-air pressure and consequent vaporization that can adversely impact visual clarity. Protracted use of high pressure can increase the risk of optic nerve damage (cite).
6. Its avoidance of retinal interaction, thereby lessening the use of mechanical stress and risk of iatrogenic damage.

While symptomatic patients can be re-operated on for subretinal PFCL, long-term residual PFCL droplets may cause retinal degeneration, barrier effects, and photoreceptor toxicity, and in turn glial proliferation and vision loss [9, 10]. As such, we believe this prophylactic measure may provide surgeons peace of mind when performing PFCL clearance. Limitations of this technique are two-fold: firstly, like many fundus-implicated procedures, there remains an inability to access the retina’s most peripheral regions, rendering them beyond the scope of intervention. However, the fact that no cases exhibited subretinal PFCL suggests that droplets at risk for migration and centralized and that the adjuvant drip-rinsing may aid in efficacy. Secondly, the technique is only applicable to epiretinal PFCL, while subfoveal retained PFCL still presents the highest risks. Future studies should explore the longer-term outcomes of patients who receive this technique when undergoing PPV, as well as with an expanded inclusion criteria to include other kinds of retinal detachments. We believe this adjuvant technique for BSS drip-rinsing of droplets is a reliable, pragmatic, and safe strategy for addressing remnant epiretinal PFCL.

## Electronic supplementary material

Below is the link to the electronic supplementary material.

Supplementary Material 1

## Data Availability

No datasets were generated or analysed during the current study.

## Abbreviations

PFCL: Perfluorocarbon liquid
BSS: Basic salt solution
PPV: Pars plana vitrectomy
RRD: Rhegmatogenous retinal detachment
UWF-SLO: Ultrawide-field scanning laser ophthalmoscopy
OCT: Optical coherence tomography
